# Gut microbiome in neuropsychiatric disorders

**DOI:** 10.1590/0004-282X-ANP-2021-0052

**Published:** 2022-03-16

**Authors:** Diana Marcela MEJÍA-GRANADOS, Benjamín VILLASANA-SALAZAR, Ana Carolina COAN, Liara RIZZI, Marcio Luiz Figueredo BALTHAZAR, Alexandre Barcia de GODOI, Amanda Morato do CANTO, Douglas Cescon da ROSA, Lucas Scárdua SILVA, Rafaella do Rosario TACLA, Alfredo DAMASCENO, Amanda DONATTI, Wagner Mauad AVELAR, Alessandro SOUSA, Iscia LOPES-CENDES

**Affiliations:** 1 Universidade de Campinas, Faculdade de Ciências Médicas, Departamento de Medicina Translacional, Campinas SP, Brazil. Universidade de Campinas Faculdade de Ciências Médicas Departamento de Medicina Translacional Campinas SP Brazil; 2 Instituto Brasileiro de Neurociências e Neurotecnologia, Campinas SP, Brazil. Instituto Brasileiro de Neurociências e Neurotecnologia Campinas SP Brazil; 3 Universidad Nacional Autónoma de México, Instituto de Neurobiologia, Juriquilla, Querétaro, México. Universidad Nacional Autónoma de México Instituto de Neurobiologia Juriquilla Querétaro México; 4 Universidade de Campinas, Faculdade de Ciências Médicas, Departamento de Neurologia, Campinas SP, Brazil. Universidade de Campinas Faculdade de Ciências Médicas Departamento de Neurologia Campinas SP Brazil

**Keywords:** Gastrointestinal Microbiome, Metagenomics, Nervous System Diseases, Transplantation, Precision Medicine, Microbioma Gastrointestinal, Metagenômica, Doenças do Sistema Nervoso, Transplante, Medicina de Precisão

## Abstract

**Background:**

Neuropsychiatric disorders are a significant cause of death and disability worldwide. The mechanisms underlying these disorders include a constellation of structural, infectious, immunological, metabolic, and genetic etiologies. Advances in next-generation sequencing techniques have demonstrated that the composition of the enteric microbiome is dynamic and plays a pivotal role in host homeostasis and several diseases. The enteric microbiome acts as a key mediator in neuronal signaling via metabolic, neuroimmune, and neuroendocrine pathways.

**Objective:**

In this review, we aim to present and discuss the most current knowledge regarding the putative influence of the gut microbiome in neuropsychiatric disorders.

**Methods:**

We examined some of the preclinical and clinical evidence and therapeutic strategies associated with the manipulation of the gut microbiome.

**Results:**

targeted taxa were described and grouped from major studies to each disease.

**Conclusions:**

Understanding the complexity of these ecological interactions and their association with susceptibility and progression of acute and chronic disorders could lead to novel diagnostic biomarkers based on molecular targets. Moreover, research on the microbiome can also improve some emerging treatment choices, such as fecal transplantation, personalized probiotics, and dietary interventions, which could be used to reduce the impact of specific neuropsychiatric disorders. We expect that this knowledge will help physicians caring for patients with neuropsychiatric disorders.

## RESUMO

**Antecedentes:**

Os transtornos neuropsiquiátricos são uma importante causa de morte e invalidez no mundo. Os mecanismos subjacentes a esses transtornos incluem uma constelação de etiologias estruturais, infecciosas, imunológicas, metabólicas e genéticas. Avanços nas técnicas de sequenciamento do DNA têm demonstrado que a composição do microbioma entérico é dinâmica e desempenha um papel fundamental não apenas na homeostase do hospedeiro, mas também em várias doenças. O microbioma entérico atua como mediador na sinalização das vias metabólica, neuroimune e neuroendócrina.

**Objetivo:**

Apresentar os estudos mais recentes sobre a possível influência do microbioma intestinal nas diversas doenças neuropsiquiátricas e discutir tanto os resultados quanto a eficácia dos tratamentos que envolvem a manipulação do microbioma intestinal.

**Métodos:**

foram examinadas algumas das evidências pré-clínicas e clínicas e estratégias terapêuticas associadas à manipulação do microbioma intestinal.

**Resultados:**

os táxons-alvo foram descritos e agrupados a partir dos principais estudos para cada doença.

**Conclusões:**

Entender a fundo a complexidade das interações ecológicas no intestino e sua associação com a suscetibilidade a certas doenças agudas e crônicas pode levar ao desenvolvimento de novos biomarcadores diagnósticos com base em alvos moleculares. Além disso, o estudo do microbioma intestinal pode auxiliar na otimização de tratamentos não farmacológicos emergentes, tais como o transplante de microbiota fecal, o uso de probióticos e intervenções nutricionais personalizadas. Dessa forma, terapias alternativas poderiam ser usadas para reduzir o impacto dos transtornos neuropsiquiátricos na saúde pública. Esperamos que esse conhecimento seja útil para médicos que cuidam de pacientes com diversos transtornos neuropsiquiátricos.

## INTRODUCTION

**Figure 1.**
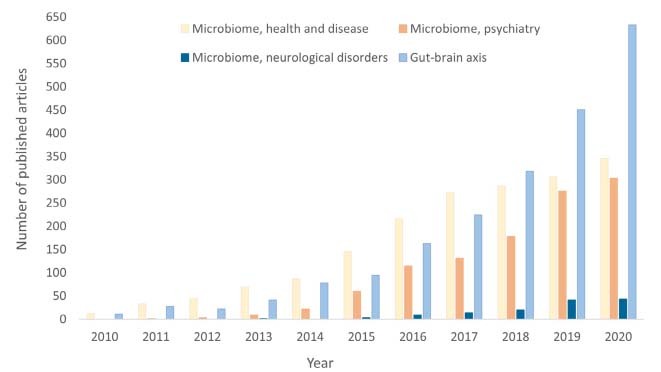
Citations in PubMed related to microbiome research over the last decade.

Over the past decade, microbiomics have emerged as a new field led by advances in culture-independent methods and next-generation sequencing technologies. These methods have provided a broader understanding of how the interaction between microbes and humans can profoundly influence host homeostasis and different disease states^1^. The literature has reflected the growing number of studies on the putative influence of the microbiome on human health and disease (Figure 1). In humans, bacteria are the most prevalent domain, and it has been estimated that the ratio of microbes to human cells in an adult is nearly 1:1^2^. The human microbiome is defined as the compendium of microbial communities (including bacteria, archaea, viruses, protozoa, and fungi) living in a given body niche^3^^,^^4^.

It has been demonstrated that the gut microbiome can be shaped by prenatal conditions, lifestyle, aging, host genetics, antibiotic use, and even geography. Moreover, it has been shown that microbial aggregates on the large intestine can modulate a wide range of host physiological processes related to immune system maturation, food metabolism, energy production, and brain development^5^^,^^6^.

The crosstalk between the gut microbiome and the brain is known as the gut microbiota-brain axis (MBA), which involves immunological, neuroendocrine, metabolic, and stress-response pathways^7^. This axis appears to be a cardinal mediator in a repertory of complex traits that range from metabolic to neuropsychiatric conditions^8^. Despite the vast contribution of animal models to elucidate biological mechanisms underlying host-microbiota interactions, there are difficulties in translating the findings in animal experiments to human research. Also, differences in methodologic standards, metadata curation, and reference databases management (https://portal.hmpdacc.org/; https://www.sanger.ac.uk/resources/downloads/bacteria/metahit/) can be potential pitfalls for study comparison, interpretation, and reproducibility^9^.

Since cohorts in microbiome studies tend to be small and heterogeneous, it is difficult to know which observations are generalizable to larger patient populations. Metabolomic, proteomic, and big data analyses of microbiome function will be critical to determine if the conclusions of these studies can apply to the clinical setting. Further experimental studies using *in vitro* or *in vivo* models are needed to understand the direct impact and causal relationships between host and microbes in order to control both known and potential hidden confounders. In this way, it still not currently possible to determine whether changes in the microbiota could be merely an epiphenomenon rather than the primary cause of the problem*.* Thus, few clinicians currently recognize that the gut microbiome is relevant to human neurophysiology because the nature of the relationship between microbiota and disease is still unclear.

In this context, we aim to present and discuss the current literature on the putative biological significance of the gut microbiome in neuropsychiatric disorders. We hope to show why this field is emerging as a possible source of therapeutic targets in these disorders and how it can be incorporated into personalized medicine strategies^4^. We will describe only the major studies related to each disease; however, additional references are presented in Tables 1, 2, 3, 4, 5.

**Table 1.** Summary of the main studies about gut microbiome in multiple sclerosis.

Title of the study	Sample size	Main findings	References
Cross-reactivity between related sequences found in *Acinetobacter sp.*, *Pseudomonas aeruginosa*, myelin basic protein, and myelin oligodendrocyte glycoprotein in multiple sclerosis	n=71 (26 patients with MS; 20 patients with unilateral hemiplegia due to a cerebrovascular accident; 25 controls)	Antibodies to mimicry peptides from *Acinetobacter*, *P. aeruginosa*, myelin basic protein, and myelin oligodendrocyte glycoprotein were significantly elevated in patients compared to controls.	Hughes et al.^23^
Gut bacteria from multiple sclerosis patients modulate human T cells and exacerbate symptoms in mouse models	n=142 (stool samples from 71 patients with MS and 71 controls)	Increased *Akkermansia muciniphila* and *Acinetobacter calcoaceticus* in patients, which induced pro-inflammatory responses. Reduced *Parabacteroides distasonis* in patients with stimulated anti-inflammatory IL-10-expressing human CD4+CD25+ T cells and IL-10+FoxP3+ Tregs in mice. Microbiota transplants from patients into germ-free mice induced more severe experimental autoimmune encephalomyelitis compared with controls.	Cekanaviciute et al.^24^
Gut microbiota in multiple sclerosis: Possible influence of immunomodulators	n=15 (7 patients with MS and eight controls)	Lower abundance of *Faecalibacterium* in patients. Different community composition in patients treated with Glatiramer acetate regarding *Bacteroidaceae*, *Faecalibacterium*, *Ruminococcus*, *Lactobacillaceae*, *Clostridium*, and other Clostridiales. Untreated patients had an increase in the *Akkermansia*, *Faecalibacterium*, and *Coprococcus* genera after vitamin D supplementation compared to the other groups.	Cantarel et al.^26^
Alterations of the human gut microbiome in multiple sclerosis	n=103 (60 patients with RRMS and 43 controls)	Increased abundances of *Prevotella* and *Sutterella* in patients using a disease-modifying treatment and decreased *Sarcina*, compared with untreated patients.	Jangi et al.^27^
Associations between the gut microbiota and host immune markers in pediatric multiple sclerosis and controls	n=24 (15 pediatric patients with RRMS and nine controls).	There was no difference in immune markers (Th2, Th17, and Tregs) between patients and controls, although there were divergent gut microbiota associations. There was a positive correlation between richness and Th17 in patients. *Bacteroidetes* were inversely associated with Th17 in patients, and *Fusobacteria* correlated with Tregs in controls.	Tremlett et al.^28^
Immunological and Clinical Effect of Diet Modulation of the Gut Microbiome in Multiple Sclerosis Patients: A Pilot Study	n=20 (20 patients with RRMS with follow-up of 12 months)	In the group with HV/LP diet compared to the western diet group, the *Lachnospiraceae* family was more abundant, IL-17-producing and PD-1-expressing T CD4+ lymphocytes were significantly decreased. The relapse rate in 12 months and the EDSS score were significantly reduced.	Saresella et al.^29^

Th2: T helper 2 cells; Th17: T helper 17 cells; Tregs: Regulatory T cells; RRMS: relapsing-remitting multiple sclerosis; MS: multiple sclerosis; HV/LP: high vegetable/low protein; EDSS: expanded disability status scale.

**Table 2.** Summary of the main studies about gut microbiome in stroke.

Species	Diagnosis (n, age)/Stroke model (n)	Biological sample	Methods	Main shifts in gut microbiota composition (genus/phylum)	References
Human	-531 Finnish males -45-70 years old -From the general population of the METabolic Syndrome In Men (METSIM) study	-Stool -Fasting blood samples	-16S rRNA gene amplification -NMR spectroscopy -liquid chromatography with on-line tandem mass spectrometry (LC-MS/MS)	-↑ Prevotella and Peptococcaceae were associated with ↑ plasmatic TMAO -↑ Unclassified Clostridales was associated with ↑ plasmatic TMAO and ↑ glutamine levels -↑ *Tenericutes* and *Christensenellaceae* were associated with ↑ acetate levels, ↑ HDL levels, ↓ BMI, and ↓ TG -↑ *Christensenellaceae* associated with ↓ leucine and ↓ isoleucine -↑ *Blautia* and *Dorea* were associated with high serum levels of glycerol, monounsaturated fatty acids, and saturated fatty acids -↑ *Methanobacteriaceae* and *Coprococcus* was associated with lower levels of TGs	Org et al.^37^
Human	-349 Dutch ischemic and hemorrhagic stroke patients, median age 71 years -Collected within 24 h of hospital admission -51 Dutch outpatient age-and sex-matched non-stroke controls, median age 72 years	-Plasma protein biomarkers -Rectal swabs	-H-NMR spectroscopy -LC-MS -16S rRNA amplicon sequencing	-↑ *Escherichia/Shigella, Peptoniphilus*, *Ezakiella*, and *Enterococcus* (potentially invasive aerobic bacterial genera) in patients with ischemic stroke and cerebral hemorrhage. -↑ *Blautia, Subdoligranulum*, and *Bacteroides* in controls and patients with a TIA. -Stroke patients displayed a higher prevalence of TMA-producing bacteria and lower plasma levels of TMAO -Loss of butyrate-producing bacteria in stroke patients	Haak et al.^38^
Human	-30 Chinese patients with cerebral ischemic stroke (21 males, nine females) -30 healthy Chinese control (18 males, 12 females) -Mean age in CI was 60.47±10.57 vs. 64.17±12.67 in the control group	-Fecal samples -Blood levels of HDL, LDL, GLUC, UA, TG, and HCY	-Amplification of the V1-V2 region of the 16S rRNA gene	-↑ Short-chain fatty acids producers such as *Odoribacter*, *Akkermansia*, *Ruminococcaceae_UCG_005*, *norank_p_Flavobacteriaceae*, *norank_p_Parcubacteria,* and *Victivallis* in the CI group.	Li et al.^39^
Human	-12 Swedish patients with symptomatic atherosclerotic plaques (who had undergone carotid endarterectomy for minor ischemic stroke, transient ischemic attack or amaurosis fugax) -13 Swedish sex-and age-matched controls.	-Stool samples -Serum b-Carotene and lycopene	-Shotgun metagenomics.	-↑ *Collinsella* was enriched in patients with symptomatic atherosclerosis -Genes encoding peptidoglycan synthesis were enriched in patients -Depletion of genes encoding phytoene dehydrogenase in patients -↓ b-carotene (antioxidant) in patients	Karlsson et al.^41^
C57BL/6J and Rag1/ mice	-Two distinct models of acute MCA occlusion: fMCAo and fMCAo	-Mice feces	-16S rRNA amplicon	-↓ Species diversity and bacterial overgrowth of *bacteroidetes* were identified as hallmarks of poststroke dysbiosis -Dysbiotic microbiome induces: ↓ intestinal motility, intestinal barrier dysfunction, a pro-inflammatory T-cell polarization in the intestine, and ischemic brain -Therapeutic FMT improves stroke outcome	Singh et al.^42^
Human	-Chinese patients with large-artery atherosclerotic ischemic stroke and TIA (322 provided blood samples and 141 provided fecal samples). -Chinese asymptomatic controls (231 provided blood samples and 94 provided fecal samples). -18 to 80 years	-Blood TMAO levels -Fresh fecal sample	-LC-MS -Pyrosequencing of 16S rRNA tags	-↑ Opportunistic pathogens, such as *Enterobacter*, *Megasphaera*, *Oscillibacter*, and *Desulfovibrio* in stroke and transient ischemic attack group. -↓ Commensal or beneficial genera including *Bacteroides, Prevotella*, *and Faecalibacterium* in patients group. -↓ TMAO level in the stroke and transient ischemic attack patients	Yin et al.^45^
Human (males) and C57BL/6 mice	-Stroke cohort: 104 Chinese patients with acute ischemic stroke and 90 healthy Chinese individuals -Validation cohort: 83 Chinese patients and 70 healthy Chinese individuals -18 to 80 years. -Experimental Stroke MCA occlusion (mice) -Stroke Dysbiosis Index (SDI) model	-Fecal samples	-16S rRNA gene V4 region	-↑ *Butyricimonas*, *Un Rikenellaceae*, *Un Ruminococcaceae*, *Oscillospira*, *Bilophila*, *Un Enterobacteriaceae* and *Parabacteroides* and ↓ *Fecalibacterium*, *Clostridiaceae*, and *Lachnospira* in patients with higher SDI -SDI was significantly higher in stroke patients than in healthy controls -A higher SDI increased the probability of unfavorable outcomes. -Mice that received FMT from high-SDI patients developed severe brain injury with elevated IL-17+ γδ T cells in the gut compared to mice receiving FMT from low-SDI patients	Xia et al.^46^

TMA: trimethylamine; TMAO: trimethylamine N-oxide; BMI: body mass index; TG: triglycerides; HDL: high-density lipoprotein; MCA: middle cerebral artery; fMCAo: post-filament MCA occlusion model; cMCAo: permanent distal MCA occlusion model; FMT: fecal microbiota transplantation; TIA: transient ischemic attack; H-NMR: proton nuclear magnetic resonance. Un: unclassified. SDI: Stroke Dysbiosis Index. CI: cerebral ischemic stroke. HDL: high-density lipoprotein; LDL: low-density lipoprotein; GLU: blood glucose; UA: uric acid; TG: triglycerides; HCY: homocysteine.

**Table 3.** Summary of main studies about gut microbiome and dementias.

Species	Diagnosis (n, age)	Intervention	Biological Sample	Methods	Main shifts in gut microbiota composition (genus/phylum)/Outcome	Reference
Human	-25 American AD patients and 88 asymptomatic control group. -Age-and sex-matched (mean ±70.3)	NA	-Fecal samples -CSF biomarkers included Aβ42/Aβ40, phosphorylated tau (p-tau), the ratio of p-tau/Aβ42, and chitinase-3-like protein 1 (YKL-40)	-16S rRNA sequencing (V4)	-Decreased richness and diversity in the AD group -↑ In the phylum Bacteroidetes and Bacteroides at genus level in the AD group -↓ Actinobacteria phylum and Bifidobacterium genus in the AD group. -Significant associations between CSF biomarker YKL-40 and abundance of *Bacteroides*, *Turicibacter*, and *SMB53* (family *Clostridiaceae*) in the AD group	Vogt et al.^52^
Human	-97 Chinese subjects: 33 AD, 32 aMCI, and 32 HC -Aged 50 to 85 years	NA	-Fecal samples	16S rRNA Miseq sequencing (V3-V4 region)	-↓ Microbial diversity in the AD group -*Proteobacteria* was highly enriched in the AD group -The models based on the abundance of family *Enterobacteriaceae* could distinguish AD from both aMCI (AUC=0.688) and HC (AUC=0.698)	Liu et al.^53^
Human	-17 American participants (11 had mild cognitive impairment and 6 were cognitively normal) -Age: 64.6±6.4 years	-MMKD versus for 6-weeks separated by 6-weeks washout periods	-Fecal samples -Short-chain fatty acids (SCFAs), Aβ-40, Aß-42, total tau, and Tau-p181 before and after diet interventions	-16S rRNA gene sequencing (V4 region) -HPLC	-↑ Abundance of Proteobacteria in MCI group -At the family level MCI group had ↑ abundance of *Enterobacteriaceae* and *Mogibacteriaceae* -↑ Abundance of genera *Phascolarctobacterium* and *Coprococcus* in MCI group. -Proteobacteria correlate positively with the Aβ-42/Aβ-40 ratio in the MCI group -↑ Abundance of *Enterobacteriaceae, Akkermansia, Slackia, Christensenellaceae,* and *Erysipelotriaceae* on MMKD -MMKD slightly reduces fecal lactate and acetate while increasing propionate and butyrate	Nagpal et al.^56^

AD: Alzheimer’s disease; aMCI: amnestic mild cognitive impairment; MCI: mild cognitive impairment; HC: healthy controls, CN: cognitively normal; AUC: area under the ROC curve; CSF: cerebrospinal fluid; YKL-40: chitinase-3-like protein 1; MMKD: Mediterranean-ketogenic diet; AHAD: American Heart Association Diet; SCFAs: Short-chain fatty acids; Aβ: Amyloid β; Tau-p181: phosphorylated tau 181; HPLC: high-performance liquid chromatography; NA: not applicable.

**Table 4.** Summary of the main studies about gut microbiome in epilepsy.

Species	Diagnosis (n, age)/Epilepsy model (n)	Intervention	Biological Sample	Methods	Main shifts in gut microbiota composition (genus/phylum)/Outcome	Reference
Swiss Webster mice	-6-Hz-induced seizure model of refractory epilepsy -Kcna1 ^-/-^ mouse model for TLE and SUDEP -3-4-week-old male and female mice	KD for 14 days	-Fecal and blood samples -FMT	-16S rDNA profiling -Metabolomics -Bacterial FISH	-↑ Relative abundance of *Akkermansia muciniphila* and *Parabacteroides* *-* ↑ GABA/glutamate in the hippocampus -↓ γ-glutamylated amino acids in both colonic lumenal content and sera	Olson et al.^61^
Human	-12 Swedish children with drug-resistant epilepsy, 3 to 15 years old -11 healthy Swedish controls (patients’ parents)	-Classic KD for three months	-Blood levels of glucose and β-hydroxybutyric acid -Fecal samples	Shotgun metagenomic	-↓ *Bifidobacterial*, *Actinobacteria*, *E. rectale* and *Dialister* -↑ Relative abundance of *E. coli*	Lindefeldt et al.^62^
Human	-20 Chinese patients with refractory epilepsy -14 males, six females -Median age 4.2 years	-Classic KD for six months	-Fecal samples -Blood glucose and ketones levels	-V3 and V4 amplification of the 16S rRNA gene	-Overall, KD reduced gut biodiversity -↓ Abundance of Firmicutes and Actinobacteria and ↑ Bacteroides -*Clostridiales, Ruminococcaceae, Rikenellaceae, Lachnospiraceae,* and *Alistipes* were enriched in the refractory group	Zhang et al.^63^
Human	-14 Chinese children with refractory epilepsy-30 age-matched healthy infants	-Classic KD for one week	-Fecal samples	16S rRNA sequencing	-64% of epileptic infants improved (50% decrease in seizure frequency) -↑ *Bacteroides, Prevotella,* and *Bifidobacterium* after treatment. -↓ *Proteobacteria* after KD	Xie et al.^64^
Human	-8 Korean children with refractory epilepsy aged 1 to 7 years -32 age-matched healthy controls	NA	-Fecal samples	-16S rRNA gene (V3-V4) sequencing	-↓ *Bacteroidetes, Proteobacteria* and ↑ *Actinobacteria* in epileptic group -↓ Microbial richness in epileptic patients. -Biomarkers for refractory epilepsy were: *Enterococcus faecium, Bifidobacterium longum,* and *Eggerthella lenta*	Lee et al.^66^
Human	-30 Turkey patients with idiopathic focal epilepsy (16 men, 14 women, mean age of 41.3 years) -10 healthy controls (mean age 31.7)	NA	-Fresh stool samples	-16s rDNA (V3-V4) sequencing	-↑*Proteobacteria* in patients -↑*Campylobacter, Delftia, Haemophilus, Lautropia, Neisseria* in IFE group	Şafak et al.^67^
Human	-Chinese patients (males and females) with epilepsy -Drug-resistant (n=42) -Drug-sensitive (n=49) -65 healthy controls -5 to 50 years old	NA	-Fecal samples	-V3-V4 amplification of the 16S rRNA gene	-↑ Abundance of *Verrucomicrobia* and rare microbiota in patients with DRE -↑ *Bifidobacteria* and *Lactobacillus* in patients with ≤4 seizures per year	Peng et al.^68^
Human	-Chinese participants. -Exploration cohort: 55 patients and 46 controls -Validation cohort 13 patients and ten controls -Ages ranged from 15 to 60 years -Create a model to distinguish DRE from DSE	NA	-Fecal samples	-16S rRNA (V3-V4) sequencing	-↓ Alfa diversity in patients -At the phylum level, patients had: ↑ *Actinobacteria* and *Verrucomicrobia,* and ↓ *Proteobacteria* *-A*t the genus level, patients demonstrated: ↑ *Prevotella_9*, *Blautia,* and *Bifidobacterium* -The phylum *Cyanobacteria* and genus *Parabacteroides* were depleted in the DRE group *-* Fecal microbiota could serve as a potential biomarker for disease diagnosis	Gong et al.^69^
Human	-Pilot study -45 patients with DRE -Mean age 44 years	A mixture of 8 bacterial species (probiotics) daily for four months	Levels of cD-14, interleukin 6, and γ-aminobutyric acid were analyzed	NA	28.9% of all patients displayed a greater than 50% reduction in the number of seizures	Gómez-Eguílaz et al.^70^
Human	-6 Dutch patients with DRE -10-16 years old	-Antibiotics exposure	NA	NA	-Patients without seizures (short-term) during antibiotic treatment	Braakman and van Ingen^71^

TLE: temporal lobe epilepsy; SUDEP: sudden unexpected death in epilepsy; FISH: Bacterial Fluorescence *In Situ* Hybridization; KD: ketogenic diet; DRE: drug-resistant epilepsy; FMT: fecal microbiota transplantation; DRE: drug-resistant epilepsy; DSE: drug-sensitive epilepsy; IFE: idiopathic focal epilepsy NA: not applicable.

**Table 5.** Summary of the main studies about gut microbiome in Parkinson’s disease.

Species	Diagnosis (n, age)	Intervention	Biological Sample	Methodology	Main shifts in gut microbiota composition (genus/phylum)/Outcome	Reference
Human	-55 Finnish patients with PD (mean age 67.63±5.21 years) -56 controls (mean age 66.38±6.73 years)	NA	-Fecal and plasma samples	-V3-V4 regions of the 16S rRNA gene -Gas chromatograph	-↑ Calprotectin and ↓ SCFAs in the stool of participants with PD (sex-dependent manner) -Fecal zonulin correlated positively with fecal NGAL -Butyric acid levels were higher in PD patients with the *Prevotella* enterotype	Aho et al.^74^
Human	-64 Finnish patients with Parkinson’s (mean age 65.2±5.52) -64 Finnish control individuals (mean age 64.45±6.9)	NA	-Fecal samples	16S rRNA gene sequencing (V3-V4 region)	-Progressed PD patients had a Firmicutes-dominated enterotype more often than stable patients or control subjects -↓ Abundance of *Prevotella* in faster-progressing PD patients	Aho et al.^75^
	-Samples were collected twice, on average 2.5 years apart					
Human	-51 Chinese PD patients (mean age 62.4±8.2 years) -48 age-matched, healthy controls (mean age 62.2±9.2 years)	NA	-Fecal samples	-16S-rRNA gene sequencing (V4 region)	-PD patients showed decreased species richness, phylogenetic diversity, β-diversity, and altered relative abundance in several taxa -↑ *Akkermansia* and ↓ *Lactobacillus* in PD patients	Li et al.^76^
Human	-72 Finnish PD patients (mean age 65.3±5.5) -72 control subjects (mean age 64.5±6.9)	NA	-Fecal samples	-16S rRNA gene Pyrosequencing (V1-V3 regions)	-↓ Abundance of *Prevotellaceae* in feces of PD patients (by 77.6% as compared with controls) -The relative abundance of *Enterobacteriaceae* was positively associated with the severity of postural instability and gait difficulty	Scheperjans et al.^77^

PD: Parkinson’s disease; SCFAs: short-chain fatty acids; NGAL: neutrophil gelatinase-associated lipocalin; NA: not applicable.

## GUT MICROBIOME STRUCTURE AND FUNCTION: INTESTINAL AND SYSTEMIC IMPACT

The gastrointestinal (GI) tract is considered the organ that harbors the highest amount of commensal microorganisms, reaching 10^13^ bacteria/mL^2^. Bacteroidetes, Firmicutes, Actinobacteria, Verrumicrobia, Proteobacteria, and Fusobacteria have been identified as the core enterotypes at the phylum level, which consist of more than 1000 species^3^. These consortia tend to remain steady throughout adult life, but preclinical evidence in both animals and humans have revealed that pre-and post-natal colonization of the gut has specific signatures and interindividual variations (~20%), depending on critical events such as maternal conditions, perinatal infections, mode of delivery, breastfeeding, diet, antibiotic exposure, and host genetics^5^^,^^6^.

Different approaches comparing germ-free mice and conventionally colonized controls demonstrated that the abnormal composition of the gut microbiota led to functional and structural changes in the gastrointestinal tract. A variety of morphological defects on mucosa-associated lymphoid tissue and intestinal microvasculature, including reducing the number of Paneth cells, Peyer’s patches, and villi size, were already described in mice models^10^. Similarly, the loss of microbial diversity can negatively affect the expression of genes related to nutrient absorption, mucosal cellularity, and barrier fortification^11^. *In vitro* and *in vivo* studies have examined the importance of Toll-like receptors (TLRs) present on the surface of intestinal epithelial cells and immune T cells^12^. The signaling transmission mediated by TLRs can also modulate mechanisms encompassing the host’s tolerance to commensal bacteria and the inhibition of pro-inflammatory cytokines such as the tumor necrosis factor (TNF), interleukin-6 (IL-6), and interleukin-1B (IL-1B)^13^. These findings have been classically associated with several GI disorders, including inflammatory bowel disease (IBD), Crohn’s disease (CD), and ulcerative colitis (UC), as well as irritable bowel syndrome (IBS), functional dyspepsia (FD), and colorectal cancer (CRC)^14^^,^^15^. Besides, observational studies have indicated that the loss of beneficial microorganisms affects ecological interactions among local populations and drive systemic diseases. Some of the metabolic processes that are impaired in patients with obesity, diabetes mellitus, and non-alcoholic fatty liver disease are associated with the synthesis of vitamins, xenobiotic transformation, and bacteria-derived molecules production, including secondary bile acids and short-chain fatty acids (SCFA) production^16^^,^^17^.

## INTESTINAL MICROBIOTA AFFECTS BRAIN FUNCTION AND HAS IMMUNOMODULATORY PROPERTIES

The human gut contains its own neural system, consisting of more than one hundred million neuron cells (10^8^)^18^. Crosstalk between the central nervous system (CNS) and the gut microbiome is known as the gut microbiota-brain axis, and it is orchestrated at different anatomical levels. It occurs through an intricate network of afferent and efferent circuits alongside the vagus nerve, enteric nervous system, and hypothalamic-pituitary-adrenal axis. Thus, the gut connectome integrates neuroendocrine, enteroendocrine, neuroimmune, and metabolic signaling pathways responsible for regulating functions associated with digestion, tract motility, and brain development. Furthermore, gut microbes can control host responses to vascular injury and autoimmunity by modifications in both the blood-brain barrier (BBB) and brain lymphatic system^7^^,^^15^.

Additionally, evidence from intervention studies in germ-free mice, where a diverse microbiota is absent, has also shown the great potential that microorganisms have in regulating microglia differentiation and maturation. Other features commonly displayed in gnotobiotic models are the abnormal expression of proteins related to synaptic plasticity, such as the brain-derived neurotrophic factor and the impairment in global cognition responses. For instance, in a study carried out by Möhle et al., the authors pointed out that adult hippocampal neurogenesis and global cognition responses can be restored after oral supplementation with probiotics via expansion of Ly6C+ monocytes^19^^,^^20^. Notably, it has been reported that microbial metabolites such as SCFAs, secondary bile acids, precursors of the lipid biosynthesis (propionate), and specific amino acids (tryptophan) are critical in modulating the release of host cytokines and hormones such as peptide YY, vasoactive intestinal peptide, glucagon-like peptide-1 (GLP-1), and melatonin, as well as neurotransmitters such as serotonin (5-HT), catecholamines, and gamma-aminobutyric acid (GABA). All of these affect brain functions related to feeding, stress response, emotional behaviors, aging, and cognition^17^^,^^21^. Furthermore, intestinal dysbiosis appears to be linked to the development of brain autoimmunity driven by specific T cell subtypes, pro-inflammatory cytokines, endothelial barrier impairment, and neurodegeneration^7^^,^^22^. The next section will explore some of the neuropsychiatric disorders thought to be influenced by microbiome disturbances.

## MULTIPLE SCLEROSIS

Previous studies have linked T helper 17 cells (Th17) to MS pathogenesis through its effect in exacerbating experimental autoimmune encephalomyelitis (EAE). Regulatory T cells (Treg) have an essential role in suppressing inflammation in the CNS in EAE models^22^^,^^23^. Cekanaviciute et al. have identified increased EAE disease scores and deficient IL-10+ Treg induction in mice colonized with microbiota from patients with MS^24^. Furthermore, some butyrate-producing bacteria, mainly belonging to the Firmicutes phylum, have also been implicated in the pathogenesis of MS. Butyrate is known to inhibit pro-inflammatory pathways and prevent systemic exposure to intestinal antigens^25^. Bacteria, such as *Faecalibacterium* from the Firmicutes phylum, were found to be reduced in MS patients^26^.

In a study of adult patients, Jangi et al. found a higher abundance of *Methanobrevibacter* and *Akkermansia* with a lower prevalence of *Prevotella*, *Butyricimonas*, *Colinsella*, and *Slackia* in patients with MS compared to healthy controls. Combining microbiome results with the immunogenetics characteristics of patients with MS, they found a positive correlation between *Methanobrevibacter* and *Akkermansia* and a negative correlation of *Butyricimonas* with *MAPK14*, *MAPK1*, *LTBR*, *STAT5B*, *CASP1*, and HLA-DRB1 -genes associated with potentiation of the immune response in MS^27^.

Tremlett et al. studied the microbiome in pediatric MS. A phylum-level analysis found a negative association between Bacteroidetes and CD4+ T cells and Tregs and a positive association between Actinobacteria and CD4+ T cells and Tr1 (IL-10), which represent some of the most common bacterial phyla of the human microbiota. The evenness of the gut microbiome also had a strong and negative association with Th17 and T helper 2 (Th2) response in the control group^28^.

More recently, the interaction between microbiome and diet in MS has attracted attention. A study found an association between a lower number of relapses and a lower disability status scale after one year of a high vegetable/low protein diet (HV/LP diet) when compared with a classical Western diet characterized by regular consumption of red meat, processed meat, refined grains, sweetened food, salt, and saturated and omega-6 fatty acids. The HV/LP group had higher levels of bacteria from the *Lachnospiraceae* genus, and they were positively related to Treg cells^29^. Table 1 summarizes the most relevant microbiome findings in patients with MS.

## AUTOIMMUNE ENCEPHALITIS

Immuno-mediated encephalitis is an emergent group of syndromes characterized by the development of acute or subacute progressive encephalopathy (less than three months onset) that occurs due to an abnormal antibody response against cell-surface, intracellular synaptic, or intraneuronal (nuclear or cytoplasmic) antigens^30^^,^^31^.

Most of what is known about the impact of intestinal dysbiosis in patients with autoimmune encephalitis (AE) comes from studies conducted in rodents. It has been suggested that changes in the intestinal microbiota could increase the susceptibility to AE through different mechanisms. Thus, increases in abundance and richness of specific pathobionts can provoke pro-inflammatory T cell cross-reactivity due to molecular similarities with neural proteins^22^. Microbiome products of diet fermentation such as the SCFAs have a major impact on gene expression of transcription factors via epigenetic mechanisms. Indeed, in a case-control study conducted by Gong et al., fecal samples were examined in 30 patients at different phases of anti-N-methyl-D-aspartate receptor (NMDAR) encephalitis. Patients in the acute phase had low Firmicutes to Bacteroidetes (F/B) ratios than the control group^32^. These results support the theory that an imbalance in commensal microbes negatively impacts the production of SCFAs. Also, main SCFAs such as acetate (C2), propionate (C3), and butyrate (C4) can alter a variety of cellular mechanisms involving the activity of G-protein coupled receptors (GPRs), the inhibition of histones deacetylases (HDACs), and nuclear factor-jB (NF-jB), and the biosynthesis of retinoic acid, which are all essential in maintaining Treg differentiation and hence reducing neuroinflammation^33^^,^^34^.

Enrichment of the genus *Fusobacterium* was also reported in the anti-NMDAR AE. Fusobacteria species comprise gram-negative anaerobic bacilli, which are considered normal microbiota in oral cavity, GI, and urogenital tract. However, recent studies report that *F. nucleatum* can present pathogenic properties that have been implicated in oral and extraoral diseases, including neurological disorders^32^. It is believed that adhesion and invasion via hematogenous translocation are the primary strategies used by *F. nucleatum* to activate inflammatory and oncogenic genes, thus contributing to disease development and progression^35^.

## MICROBIOME AND STROKE

Studies have shown that up to 50% of patients with stroke suffer from GI complications, which has a strong association with patient recovery, deterioration of neurological functions, and mortality^36^. These features regarding stroke make it an interesting condition to look for associations between brain and gut microbiota. Indeed, several studies have shown a link between stroke outcomes and microbiota regulation of the immune system and metabolism^37^^,^^38^^,^^39^ (Table 2).

The bidirectional communication between the brain and gut after stroke involves the vagus nerve, release of damage-associated molecular patterns (DAMPs), cytokines from the injury site of the brain and gut, and migration of inflammatory or immune cells from the gut to the injury site^40^. This communication occurs by complex signaling pathways from the vagus nerve to the enteric nervous system, the neuronal-glial-endothelial interactions, and DAMPs and cytokines-induced activation of gut inflammatory and immune cells^40^.

Several studies show significant changes in the microbial diversity in fecal samples of patients after an ischemic stroke, leading to gut alterations including dysbiosis, dysmotility, hemorrhage, and sepsis^40^^,^^41^. Furthermore, Singh et al. showed a reduction in microbiota diversity after stroke events that might be associated with stress response, impaired motility, and tissue necrosis^42^. These changes may cause gut permeability modifications and the increase of circulating lipopolysaccharides (LPS) molecules, which may influence systemic inflammation and immune response after stroke.

Circulating IL-17 released by γδ T cells and IL-10 released by regulatory T cells (Tregs) were associated with increased ischemic brain injury and neuroprotective properties, respectively, after ischemic brain injury^43^. There is some evidence that these inflammatory molecules are regulated by gut microbiota, promoting a strong interrelation between brain and gut and influencing several neurological diseases^40^^,^^43^. After a stroke, dysbiosis leads to an imbalance of T-cell subpopulations (Th1, Th2, Th9, Th17, Treg, and follicular T helper cells) that trigger several types of autoimmune and inflammatory disease^40^. For example, Th1 (production of IL-2 and interferon-gamma) and Th2 (IL-4, IL-5, and IL-13) induce inflammation; Th9 (IL-9 and TGF-β) and Treg (IL-10 and IL-35) have a neuroprotective function; Th17 activates matrix metalloproteinases and causes blood-brain barrier breakdown by secreting IL-17A, IL17-F, and IL-22^40^. Benakis et al. demonstrated that antibiotic-treatment-induced dysbiosis could influence stroke outcome in *vivo* models by regulating T cells in the small intestine^43^. Some studies also observed improved stroke outcomes by fecal microbiota transplantation to control post-stroke dysbiosis^43^. Most interestingly, treatments involving reduction of *Clostridiaceae* and *S24-7 spp*. showed to be relevant to neuroprotection after stroke in mice^43^.

Zeng et al. recently raised the possibility that the microbiome might be a novel risk factor for stroke^44^. With a risk stratification approach and comparing higher-versus lower-risk patients, they found an increased risk of stroke associated with enrichment of opportunistic pathogens (e.g., *Proteobacteria*, *Bacilli*, *Enterobacteriaceae*), low abundance of butyrate-producing bacteria (e.g., *Lachnospiraceae, Ruminococcaceae*), and reduced concentrations of fecal butyrate^44^^,^^45^.

Xia et al. developed a stroke dysbiosis index (SDI) based on the patient’s gut taxonomic differences compared to healthy individuals^46^. They observed that high SDI values predicted severe brain injury in patients with stroke. Furthermore, to investigate a putative causal effect of intestinal dysbiosis, the authors performed experiments using a middle cerebral artery occlusion model in animals colonized with microbiota from affected individuals. They observed that mice transplanted with intestinal microbiota from high-SDI patients also developed an exacerbated inflammatory response, hence, worsening the acute brain injury associated with stroke^46^.

Although the results are encouraging, as described above, additional studies with larger samples and different ethnic backgrounds are needed to validate these findings. However, if further confirmed, a careful approach, including microbiome screening as a possible preventive target for stroke management is needed.

## GUT MICROBIOTA: THE RELATIONSHIP WITH DEMENTIA

Studies in germ-free animals exposed to microbial infections, human post-mortem brain samples, and microbiome analysis of living humans have revealed that disorder of the gut microbiota may underlie the development or exacerbation of Alzheimer’s disease (AD) pathology^47^. Also, available data suggest that the gut microbiota in AD is characterized by a substantial reduction in beneficial microbial diversity and presence of pathogenic species such as Proteobacteria phylum, especially the *Enterobacteriaceae* family^48^. These shifts in microbial diversity may activate immune cells and stimulate overproduction of toxic metabolites or pro-inflammatory cytokines, which contribute to the destruction of the GI mucosa. It is well known that chronic inflammation and immune dysregulation precede cognitive decline by years^49^. Increased inflammation makes gut microorganisms move from the GI tract through cells overlying the Peyer’s patches into blood and other tissues (a process known as atopobiosis)^47^. Likewise, systemic inflammation can increase BBB permeability, exposing the brain to cytokines that can lead to neuroinflammation and neuronal cell death, promoting neurodegenerative diseases^48^^,^^50^. Initially, the brain can resist, but the regenerative capacity, together with the microglia’s ability to clear toxic metabolites, decreases with time^49^.

The outer membrane component of Gram-negative bacteria is LPS, capable of triggering systemic inflammation by increasing pro-inflammatory cytokines^48^^,^^51^. Lipopolysaccharides may also modify gut homeostasis and promote gut inflammation and permeability. The abundance of Gram-negative intestinal bacteria, such as the *Enterobacteriaceae* family in individuals with AD, results in increased translocation of LPS from the gut into the circulation, which in turn may contribute to AD pathology^52^. Indeed, a study involving post-mortem brain tissue from patients with AD showed that LPS fragments co-localized with amyloid plaques in the hippocampus and neocortex^53^.

Surprisingly, bacteria can produce their own amyloid, which maintains cellular junctions, promotes the formation of biofilms, and confers resistance against physical or immune destruction^47^. Microbial and cerebral amyloids are structurally similar and can be recognized by the same TLR2/TLR1 receptor system^50^. They might activate signaling pathways known to play a role in neurodegeneration and AD pathogenesis^50^^,^^51^. The hypothesized mechanism is that bacteria-derived amyloids leak from the GI tract and accumulate in the brain, resulting in an increase of reactive oxygen species and activation of nuclear factor-κB, which upregulates the pro-inflammatory microRNA-34a^51^. Subsequently, microRNA-34a downregulates the expression of TREM2 (triggering receptor expressed on myeloid cells 2), leading to impairment of phagocytosis and contributing to the peptide accumulation β-amyloid1-42^51^. Both amyloids and LPS are potent activators of the receptor for advanced glycation end-products (RAGE) and Toll-like receptors (TLR), and their co-activation amplifies pro-inflammatory signaling leading to sustained chronic inflammation in AD^50^^,^^51^.

Commensal microbiota produces an assortment of neuroactive molecules, such as serotonin, kynurenine, GABA, catecholamines, histamine, and acetylcholine, among others^50^. The consequence of a dysbiotic bowel in the metabolism of tryptophan and kynurenic pathways is documented in AD^50^. Gut microbes may regulate the serotonergic system directly by producing serotonin or degrading the serotonin precursor, tryptophan^49^. *Escherichia coli*, an Enterobacteriaceae member, plays an essential role in regulating production and availability of serotonin, acting as a transmitter both in the CNS and in the enteric nervous system^51^. Nevertheless, gut-derived serotonin only exerts indirect effects on brain functions. Despite that, the gut is the only source of tryptophan, derived either from the diet or microbial production. Tryptophan crosses the BBB to become available for serotonin synthesis in the brain^49^. Gulaj et al. found reduced plasma concentration of tryptophan and kynurenic acid in 34 patients with AD, suggesting that dysregulation of the kynurenine route is present in AD^54^.

*Lactobacillus* and *Bifidobacterial* genera can metabolize glutamate to produce GABA. Changes in gut microbiota might compromise the endogenous production of this inhibitory neurotransmitter^51^. Alterations in GABA signaling are linked to cognitive impairment and AD neuropathy^49^. Likewise, *Lactobacillus, Lactococcus, Streptococcus*, and *Enterococcus* may produce histamine, which acts as a neurotransmitter essential for modulating neuroinflammation through TNF-α expression in the brain^55^. Furthermore, an N-methyl-D-aspartate (NMDA)-targeting neurotoxin that was observed to be raised in AD brains may be produced by gut cyanobacteria^49^.

The role of diet in these mechanisms is still poorly understood but probably extremely important^47^. Dietary patterns similar to the Mediterranean diet and Dietary Approaches to Stop Hypertension have been associated with a reduced risk of AD^56^. In contrast, a Western-style diet represents a risk factor. The variety and composition of a diet and long-term dietary habits may influence the gut microbiota composition and shape the microbial community^51^. A newly proposed insight is that the transplantation of fecal microflora from healthy people to patients with AD can help restore the intestinal microbiota and reduce the negative impact of the dysbiotic microbiome on the gut and brain functions^47^. The influence of gut microbiota on brain function is being investigated continuously. Table 3 presents additional studies on the role of gut microbiota and dementia.

## EPILEPSY, KETOGENIC DIET, AND THE INTESTINAL MICROBIOME

About one-third of patients with epilepsy have seizures refractory to anti-seizure drugs (ASD)^57^. Non-pharmacological approaches, especially the ketogenic diet (KD), are alternatives in cases of pharmacoresistant epilepsy. The KD has been used for about one hundred years, demonstrating efficacy in reducing seizure frequency, mostly in children with difficult-to-control epilepsy^58^.

The KD appears to be a powerful contributor in modulating downstream effects on an individual’s gut metagenomic composition and metabolomic profile^59^. In a dietary intervention study conducted by Ang et al., two cohorts composed of obese patients and mice were followed up during short-and long-term periods. In humans, a baseline diet and a KD with only 5% of carbohydrates content were used. Mice were submitted to three different diet types: low-fat diet (LFD), high-fat diet (HFD), and KD. Results from a 16S amplicon-based metagenomic approach of stool samples and metabolomic profile in humans demonstrated a significant reduction in the relative abundance of Actinobacteria phylum and a marked decrease in different bifidobacterial species, suggesting that the KD is sufficient to shift the gut microbiota composition. Similar findings were seen in a mice model, where ketone bodies like βHB had a direct effect in suppressing microbial proliferation of bifidobacteria. Moreover, fecal microbial transplantation from patients into germ-free mice demonstrated that the colonization of KD-associated microorganisms drove the reduction of selected members of *Bifidobacterium adolescentis*, therefore modulating the induction of Th17 cells. These findings reveal a separate pathway whereby carbohydrate restriction, rather than high-fat intake, is the main contributor to gut microbiome structure and immune response^60^.

Olson et al. demonstrated that the taxonomic composition of the gut microbiome in mice is altered after treatment with KD. They also raised the possibility that some KD-associated species such as *Akkermansia muciniphila* and *Parabacteroides merdae* and particular molecules predicted seizure protection with high accuracy and were necessary to reduce brain electrical activity^61^. Taken together, these findings are likely to be correlated with a decrease in the metabolism of ketogenic gamma-glutamylated (GG) amino acids and, therefore, to low concentrations of these amino acids in the colon, blood, and CNS. Likewise, this phenomenon can boost GABA bioavailability on the hippocampus, increasing seizure threshold in mice and contributing to the anti-seizure effect. Finally, based on these bacterial species and molecules, researchers could identify microbiome-based treatments such as microbiome transplant, live biotherapeutic products, and targeted pharmacological approaches that protect against seizures in mice^61^.

To further examine the potential of KD in shaping the intestinal microbiome in patients with epilepsy, Lindefeldt et al. analyzed the taxonomic and functional profile in children with difficult-to-control epilepsy using whole metagenomic sequencing^62^. The study consistently showed that i) there were differences in the composition of the patient’s gut regarding healthy controls before starting the intervention; ii) those differences were reflected in the reduction of relative abundances of butyrate-producing organisms such as *Eubacterium rectale* and *Dialister* during and after treatment with KD where variations were more noticeable; iii) KD decreased *Bifidobacterium* and *Dialister* had an impact not only on the production of non-digestible carbohydrates (e.g., lactate) but also on the final conversion to SCFAs (mainly acetate), which are crucial in brain physiology; finally, iv) shifts on gut microbiome associated with KD promoted the growth of *Escherichia coli*, which could trigger gut inflammation in epileptic patients^62^. These results are in line with a study carried out by Zhang et al., who explored the potential of microbial biomarkers in patients with refractory epilepsy who followed a KD for six months^63^. Overall, they observed low diversity and richness ratios in individuals undergoing a KD as well as an increase in the relative abundance of Bacteroidetes and reduction of Firmicutes and Actinobacteria phyla. They also reported a high abundance of *Clostridia* class organisms in non-responders. This class has been associated with tryptophan catabolites, which in turn are responsible for hormone secretion, neurotransmission, gut motility and permeability, and anti-oxidative effects^63^. Another work involving epilepsy and KD had a similar outcome and reported significant gut dysbiosis in the refractory group. Nonetheless, the enterotype Bacteroidetes was accumulated in both the healthy and epileptic groups after at least one week of high-fat diet therapy. This phylum has been related to seizure modulation by secretion of inflammatory cytokines, including IL6 and IL17. Also, *Cronobacter* was the predominant genus identified only in affected children, which decreased over the treatment period^64^.

It has been observed that patients with epilepsy are presumably prone to gut dysbiosis and, hence, to chronic inflammation of the intestinal epithelium^65^^,^^66^^,^^67^. In a cohort of 91 individuals, Peng et al. revealed that patients with more than four seizures per year had a predominance of *Ruminococcus* and rare bacteria genera compared to a drug-sensitive group. These findings lead to various hypotheses: i) the use of several antiepileptic drugs (AED) induces intestinal dysbiosis; ii) the prevalence of rare microorganisms modulate metabolic pathways involving ABC transporters, therefore conferring chemoresistance to the treatment; iii) *Bifidobacterial* and *Lactobacilli* genera stimulate the production of GABA and are prevalent in the drug-sensitive group^68^.

So far, all of the studies involving different epilepsy phenotypes either in humans or mice report changes in the intestinal microbiome at baseline or after a KD^69^. Still, there is little overlap across these studies in the exact microbial signatures that have been identified (Table 4). Thus, additional high-powered and well-controlled studies are needed to explore the issue better and propose new treatment options^70^^,^^71^.

## PARKINSON’S DISEASE AND MICROBIOME

It is well known that patients with Parkinson’s disease (PD) present severe non-motor symptoms at the prodromal phase of the disease, which are determinants of the quality of life in these individuals. Sensorial, neuropsychiatric, sleep dysfunction, and GI symptoms (constipation) are the most common phenotypes describe in this category^72^. Several studies using breath testing demonstrated that a considerable proportion of patients with PD have intestinal bacterial overgrowth and absorption issues, leading to intestinal constipation. On the other hand, 16S ribosomal RNA analysis from colonic biopsies and stool samples of adults with PD showed decreased SCFAs-producing bacteria such as *Blautia*, *Coprococcus*, and *Roseburia*^73^^,^^74^. Furthermore, depletion or increase in *Prevotella* and *Lactobacillus* genera was reported in several case-control studies performed in these patients^75^^,^^76^. This class of beneficial microorganisms is involved in BBB integrity, permeability, and neuronal inflammatory signaling^73^. Moreover, the increase in *Enterobacteriaceae* members was directly proportional to the severity of symptoms like stability, gait, and rigidity^77^. Conversely, in a study conducted in individuals with PD, the oral microbiome was analyzed. Male patients exhibited an increase in the abundance of *Prevotella*, which is considered an opportunistic pathobiont on the mouth, suggesting a strong role of these genera in periodontal disease^78^. Taken together, these findings support that modifications in bacteria density, taxonomic levels (dysbiosis), and mucin production may, in turn, boost the neuroglia system, triggering damage to the intestinal and brain barriers, leading to alpha-synuclein protein misfolding and finally aggregation. Likewise, chronic systemic exposure to LPS leads to the selective death of dopaminergic neurons in the substantia nigra^73^^,^^79^^,^^80^.

The vagus nerve (VN) has also been implicated in the pathogenesis of PD. Some researchers suggest that the VN can modulate neuroimmune and inflammatory signals either via top-down or through the microbiota-gut axis^79^. Thus, it has been proposed that the VN could transport alpha-synuclein to the CNS and vice versa. After examining 9,430 vagotomies in Swedish patients, Liu et al. demonstrated that truncal, but not selective vagotomy, had a protective effect against PD development^80^. In contrast, recent studies in mouse models mimicking motor and non-motor symptoms of early and late stages of the disease point out that changes in the immune response to gut bacteria could affect motor symptoms in PD^81^. In an experimental study performed by Sangjune et al., the authors demonstrated that pathologic species of a-synuclein could spread from the gut muscles to the brain through connections of the vagus nerve. These mice also showed neuropsychiatric symptoms, including anxiety, depression, olfactory dysfunction, and spatial learning and memory abnormalities. They also assessed another group of animals that were submitted to an injection of a-synuclein and vagotomy. The authors observed that no transmission of pathologic α-synuclein occurred in these animals, which were also free of the cardinal symptoms of PD^82^.

Several studies have been published exploring the gut microbiome in PD (Table 5). However, the need for well-designed clinical studies exploring the role of the gut microbiome in PD in the clinical setting is still lacking.

### Gut microbiota alterations in neuropsychiatric diseases

#### Autism Spectrum Disorder

Clinical observations indicate that patients with Autism Spectrum Disorder (ASD) have GI disturbances that include diarrhea, constipation, and abdominal pain^83^. Likewise, a growing number of studies have shown that patients with ASD have an altered gut microbiota composition compared to neurotypical individuals^84^. Moreover, GI disturbances strongly correlate with the severity of ASD symptoms, and GI disturbances are markedly associated with GI dysbiosis^83^^,^^84^. Therefore, it has been suggested that gut microbiota alterations may contribute to the pathogenesis of ASD(69). Although several studies have shown that adult rodents prenatally exposed to VPA, a model for ASD, exhibit gut dysbiosis^85^^,^^86^, there is a lack of evidence of a causal link between abnormal microbiota and ASD-like behaviors.

#### Schizophrenia

Patients with schizophrenia commonly have GI disturbances, such as constipation and GI hypomotility and inflammation^87^^,^^88^. A growing body of evidence indicates that altered gut microbiota may account for the GI disturbances and the severity of symptoms in schizophrenia patients suggesting a key role of the gut microbiota in promoting the pathogenesis of schizophrenia. Interestingly, this suggestion has been recently proved by Zheng and colleagues. In this study, the authors found that the gut microbiota from patients with schizophrenia induced behavioral alterations and modulated brain excitability when transferred to mice^89^. Other studies have also suggested that GI disturbances and gut microbiota alterations in schizophrenia may be related to the use of antipsychotic medication^90^. However, gut microbiota seems to be altered in patients even before medication^91^. Hence, to better understand the gut microbiota alterations in schizophrenia, experimental validation of clinical findings seems necessary. Furthermore, animal models of schizophrenia reinforce the clinical data showing that gut dysbiosis may be implicated in schizophrenia, mainly pointing to alterations in the *Firmicutes* phylum bacteria.

## ANXIETY AND DEPRESSION DISORDERS

Preclinical studies have shown that the intestinal microbiome seems to play a crucial role in the pathophysiology of both neuropsychiatric disorders. Also, fecal microbiota from patients diagnosed with anxiety and depression induce behavioral and physiological features of these disorders when transplanted to microbiota-deficient animals, including anhedonia, anxiety-like behaviors, and altered tryptophan metabolism^92^^,^^93^^,^^94^.

Park and colleagues showed that anxiety-and depression-like behaviors induced by olfactory bulbectomy (OBx) are related to colonic motility alterations and gut microbiota composition changes. In addition, the expression of hypothalamic corticotropin-hormone (CRH) was elevated in OBx mice, suggesting that GI disturbances and gut dysbiosis may be due to the recruitment of the hypothalamic-pituitary-adrenal axis^95^^,^^96^. Murakami et al. analyzed the gut microbiota composition of fecal samples from Wistar rats submitted to maternal separation when neonates. This early-life stressor leads to anxiety-and depression-like behaviors in adulthood. The authors found that maternal separation induced a specific reduction in *Bifidobacterium*, *Bacteroidetes*, and *Prevotella* genera^97^. Li et al. analyzed the gut microbiota of fecal samples from mice submitted to the chronic unpredictable mild stress (CUMS) model. They found that the gut microbiota of CUMS-treated mice exhibits drastic alterations in microbiota composition, including an increased α-diversity and changes in the abundance of specific microbial phyla, such as Verrucomicrobia and Proteobacteria. At the genus level, animals exposed to CUMS exhibit an increased abundance in *Helicobacter*, *Turicibacter*, *Parasutterella*, *Alistipes*, *Odoribacter*, and *Akkermansia*, but a decrease in *Barnesiella*, *Bifidobacterium*, *Lactobacillus*, and *Olsenella*^98^.

## FUTURE DIRECTIONS

Reports using cutting-edge technologies such as metagenomics and metabolomics are changing some of the established paradigms regarding the physiopathological mechanisms behind neuropsychiatric diseases. Using the potential of microbial profiles as biomarkers of neurological and mental health disorders may maximize the efficacy of existing therapies. Current challenges remain in establishing causation rather than association and translating basic science studies into clinical practice with the potential of targeting the microbiome for therapeutic purposes.
